# Gender differences in vision health-seeking behavior and vision health outcomes among rural Chinese schoolchildren by birth order and family size

**DOI:** 10.1186/s12939-023-01907-5

**Published:** 2023-05-13

**Authors:** Yunyun Zhang, Hongyu Guan, Yuxiu Ding, Jing Xue

**Affiliations:** Center for Experimental Economics in Education, Shaanxi Normal University, No. 620 West Chang’an Street, Chang’an District, Xi’an, 710119 Shaanxi Province China

**Keywords:** Gender difference, Health outcome, Health-seeking behavior, Family size, Birth order

## Abstract

**Background:**

The gender gap remains a major impediment in the path toward equality, and it is especially wide in low-income countries. Gender differences in health-seeking behaviors may be a factor. Family size and childbirth order are two critical factors affecting family resource allocation. This study examines gender differences in healthcare-seeking behaviors among children with visual impairment in rural China across different family structures (birth order and family size).

**Methods:**

We draw on a dataset containing 19,934 observations constructed by combining data from 252 different school-level surveys spanning two provinces. The surveys were all conducted in 2012 using uniform survey instruments and data collection protocols in randomly selected schools across western provinces in rural China. The sample children range in grades from 4 to 5. Our analysis compares rural girls with rural boys regarding vision health outcome and behavior (vision examination and correction).

**Results:**

The findings revealed that girls have worse vision than boys. Regarding vision health behaviors, girls have a lower overall vision examination rate than boys. There is no gender difference when the sample student is the only child or the youngest child in the family, but there is still a gender difference when the sample student is the oldest child in the family or the middle child in the birth order. When it comes to vision correction behavior, boys are more likely to own eyeglasses than girls are for groups of students with mild visual impairment, even when the sample student is the only child in the family. However, when the sample student has another brother or sister (the sample student is the youngest, the oldest child in the family, or the middle child in the birth order), the gender difference disappears.

**Conclusions:**

Gender differences in vision health outcomes are correlated with gender differences in vision health-seeking behaviors among rural children. Depending on the birth order and family size, gender disparities in visual health practices vary. In the future, consideration should be given to providing medical subsidies to reduce the cost of vision health behaviors and to provide information interventions to change gender inequality in households and promote equality in children’s vision health behaviors.

**Trial registration:**

The trial was approved by the Stanford University Institutional Review Board (Protocol No. ISRCTN03252665). Permission was received from local Boards of Education in each region and the principals of all schools. The principles of the Declaration of Helsinki were followed throughout. Written informed consent was obtained from at least one parent for all child participants.

## Introduction

Gender differences in health outcomes exist among children in low-income countries, with girls usually having worse health outcomes than boys, particularly among rural children [1–4]. Girls in rural China tend to have worse general physical health than boys [5–7], as well as suffer disproportionately more than boys from specific health issues, such as anemia [8], malnutrition [9], and being underweight [9–12].

The poor health status of girls may be explained by disparities in healthcare behavior between boys and girls, especially in less developed regions [13–15]. A solid literature base shows that the phenomenon may be due to rural China having traditionally valued sons over daughters [16, 17]. Families may be more likely to allocate limited resources to sons, such as boys having more seeking behavior [18], receiving higher-level treatment [19, 20], got more medical input [21, 22].

The number of children in a family [23] and the birth order [24, 25] are two factors that impact the distribution of family resources for their children. Under resource constraints, siblings naturally compete with one another. The competition for family resources among siblings becomes fiercer as the number of siblings increases, and the average share of family resources allocated decreases. Consequently, increasing the number of siblings can lead to poor access to family resources, particularly for female children with siblings [26, 27].

Moreover, when there are more children, parents are more inclined to prioritize their younger children at the expense of their older children. Therefore older females (older sisters) are more likely to experience an unequal allocation of family resources, whereas younger girls fare better [28, 29]. However, these health service-seeking behaviors and resource allocation gap occur when health cost increases.

Visual impairment among children in rural China is of particular concern regarding gender inequalities in health outcomes and healthcare-seeking behaviors. Despite having one of the highest rates of visual impairment in the world, many rural students with visual impairment do not have eyeglasses [30, 31]. According to recent research in the same area, roughly 25% of students in grades 4 and 5 have visual impairment [32]. However, research in rural China has discovered that only around a third of students who require eyeglasses have them, and even fewer use them [33]. More than 85 percent of students with visual impairment in rural China do not use eyeglasses, according to Yi et al. (2015) [34].

The high prevalence of visual impairment among children in rural China lends itself to examining the gender gap in child health outcomes and healthcare behaviors. Specifically, we detect gender differences in vision health outcomes among rural Chinese children and vision health care-seeking behaviors. We track changes in gender differences in behaviors as sibling number and birth order vary. However, to the best of our knowledge, only one study has analyzed gender differences in children's visual health and health-seeking behaviors overall [5], and no study has examined gender differences in children's vision health and seeking behaviors across different birth orders and family sizes.

The present study aims to fill these gaps in the literature by examining gender differences in healthcare-seeking behaviors among children with visual impairment in rural China across different family structures (family sizes and birth orders). Specifically, we pursue three objectives. First, we examine whether gender differences still exist in vision health outcomes in rural China across different family structures. Second, we examine whether gender differences exist in vision health-seeking behaviors in rural China across the different family structures. Finally, we investigated whether there were gender differences in vision-correcting health behaviors among children of various family structures and health statuses when the cost-related increase. To meet these objectives, this study draws on cross-sectional data for analyses.

The remainder of the paper is organized as follows. Method section describes the data collection. Study area and sampling technique section presents the results. Data collection section discusses the policy implications of the results. Personal characters section concludes.

## Method

### Study area and sampling technique

The data were collected in two adjacent western Chinese provinces: Shaanxi and Gansu. These two provinces can be considered typical of poor rural areas in northwest China. Shaanxi's GDP per capita of USD 6108 in 2012 placed 14^th^ among China's 31 provincial administrative regions and was comparable to that of the country as a whole (USD 6091) in the same year. According to the China National Statistics Yearbook in 2012, Gansu had the second-lowest GDP per capita in the country, at USD 3100 [35].

The research covered one prefecture (each including a group of seven to ten counties) in each province. Prefectures are roughly indicative of provinces. Gansu's prefecture has a population of 3.3 million, accounting for 10% of the province’s population. The GDP per capita of USD 2680 is quite close to that of the province. Shaanxi's prefecture has 3.4 million people, accounting for nearly 10% of the province’s population. While the GDP per capita is USD 13,100, this is largely attributable to mining and other extractive sectors; income per capita is close to the province average [35].

We obtained a list of all rural primary schools in each prefecture to choose the sample. We randomly selected 252 townships and then randomly selected one school per township for inclusion in the experiment. Within schools, we concentrated our data-gathering efforts (described below) on students in grades 4 and 5. One cohort was chosen randomly from each grade, and questionnaires and visual acuity tests were administered.

### Data collection

#### Personal characters

The survey was conducted in September 2012. The survey collected detailed information on students and households. A student survey was given to all students in selected grade 4 and grade 5 classes. The student survey collected information on basic background characteristics, including age, gender, math score, whether their parents worked away from home for more than six months per year, and location, which are proven to be significant predictors of vision health-seeking behavior in previous research [33, 36, 37].

Student academic performance was measured by a 30-min standardized math test. Study utilized math as the measurement of academic performance because achievement in math is more explicitly tied to learning experiences at school, as opposed to learning experiences at home (such as reading or language achievement). To make the math test results comparable across different sample schools, study conducted our own standardized mathematics test for each sample students in our survey. The test items for each sample grade were carefully designed with assistance from educators working at the local education bureau to ensure compliance with the national curriculum, and they had been used by the research team in several previous surveys to examine student academic performance in other parts of rural China. Study pre-tested the exam multiple times to ensure its relevance and that time limits were appropriate [31, 37]. When study administered the exam in the sample schools, it was timed carefully and closely proctored by trained enumerators. All test scores were then normalized according to the distribution of scores in each grade.

Left-behind children are defined as children who remain in rural areas while their parents leave to work in urban areas. Students were also asked about siblings' situations (whether they have older brothers or sisters? whether they have younger brothers or sisters?). Study divide the family types of students into the following four categories according to the situation of siblings: student is the only child in the family, student is the youngest child in the family, student is the oldest child in the family, student is the middle child in the family.

Household surveys were also given to all students, which they took home and filled out with their caregivers. The head teacher of each classroom collected the completed household forms and forwarded them to the survey team. The household survey collected information on households that children would likely have difficulty answering (e.g., parents’ education levels and the value of family assets).

#### Vision examination

A two-step vision examination was delivered to all students in randomly selected classes in all sample schools at the same time as the school survey. First, a team of two trained staff members performed visual acuity screenings using Early Treatment Diabetic Retinopathy Study (ETDRS) eye charts, which are the gold standard for precise visual acuity measurement globally [38]. Students were defined with visual impairment if their visual acuity of the better eye is less than or equal to 0.5, or 20/40.

A linear scale with constant increments is required to calculate and compare different visual acuity levels [39, 40]. LogMAR is one of the most often used continuous scales in the field of ophthalmology/optometry. The logarithm transformation is used in this scale: for example, LogMAR = log10 (MAR). The variable MAR stands for Minimum Angle of Resolution, defined as the inverse of visual acuity, e.g., MAR = 1/VA in this definition. LogMAR provides an easy-to-understand explanation of visual acuity measurements. It has a constant 0.1 increment across its scale; each increment corresponds to around one line of visual acuity loss on the ETDRS chart. The higher the LogMAR value, the worse one's vision is.

#### Vision exam and eyeglasses own

Our analysis focuses on two key variables: vision exam and eyeglasses own. Vision exam in our study is defined by the student who had a vision exam before. Specifically, we define vision exam as a binary variable taking the value of one if a student had a vision exam before the survey.

Eyeglasses ownership in our study is defined by ownership. Specifically, we define eyeglasses ownership as a binary variable taking the value of one if a student owns a pair of eyeglasses before the survey. Students diagnosed with visual impairment in vision examination were given a short survey that included questions about whether they owned eyeglasses. If a student indicated he or she did have glasses but was not wearing them, we confirmed by asking to see them. If the eyeglasses were at home, we followed up with phone calls to the caregivers.

Among 19,934 students in 252 schools, 48% were girls, 4,939 (24.3%) students with visual impairment, only 18% of students had vision exams, and 4% owned eyeglasses. Of these, 10% of students’ families with one child, 39% of students’ families with only an older brother or sister, 34% of students’ families with only a younger brother or sister, 18% of students’ families with all older and younger brother or sister (Table 1).

**Table 1:** Student characteristics *N* = 19934

Variable	Mean	Sd	Max	Min
Vision exam ( 1 = Yes, 0 = No)	0.18	0.38	1.00	0.00
Eyeglasses ownership ( 1 = Yes, 0 = No)	0.04	0.20	1.00	0.00
Visual impairment (1 = Yes, 0 = No)	0.25	0.43	1.00	0.00
Gender (1 = female, 0 = male)	0.48	0.50	1.00	0.00
Student is the only child in the family (1 = Yes, 0 = No)	0.10	0.30	1.00	0.00
Student is the youngest child in the family (1 = Yes, 0 = No)	0.39	0.49	1.00	0.00
Student is the oldest child in the family (1 = Yes, 0 = No)	0.34	0.47	1.00	0.00
Student is the middle child in the family (1 = Yes, 0 = No)	0.18	0.38	1.00	0.00
LogMAR	0.21	0.28	1.60	-0.20
Age (Years)	10.55	1.14	15.00	8.00
Math score	0.02	0.99	2.56	-2.99
Both parents have an education level above high school (1 = Yes, 0 = No)	0.18	0.38	1.00	0.00
Left behind children (1 = Yes, 0 = No)	0.12	0.33	1.00	0.00
Household assets are in the bottom 25%(1 = Yes, 0 = No)	0.25	0.43	1.00	0.00
Location (1 = Gansu, 0 = Shaanxi)	0.52	0.50	1.00	0.00

### Statistical approach

To analyze the main interest of this study, we employed the ordinary least squares (OLS) regression model to assess whether there are gender differences in the vision health outcomes and health seeking behavior and its variation across different family structures (birth order and family size) among Chinese rural students.

1 $${\mathrm{Y}}_{\mathrm{ij}}=\mathrm{\alpha }+{\upbeta }_{1}{\mathrm{Female}}_{\mathrm{i}}+{\upbeta }_{2}{\mathrm{X}}_{\mathrm{ij}}+{\upvarepsilon }_{\mathrm{ij}}$$

where ${\mathrm{Y}}_{\mathrm{ijt}}$ is a binary indicator for the vision exam or eyeglasses ownership of student i in school j. where ${Female}_{i}$ is a dummy variable indicating whether the student is female. where ${\mathrm{X}}_{\mathrm{ij}}$ represents a vector of baseline variables that would be correlated with vision care-seeking behaviors. These baseline variables include demographic factors (age, academic performance), socioeconomic and family factors (whether he/she is a left-behind child, parental education, household asset index, location), and vision-related factors (visual acuity). ${\upvarepsilon }_{\mathrm{ijt}}$ is a random error term.

To investigate gender differences in vision health-seeking behavior among students from various family structures. Regressions were run on subsamples of different family structures. In all regression models, we adjust standard errors for clustering at the school level using the cluster-corrected Huber-White estimator. All analyses were performed using Stata 15.0 (Stata Corp., Texas, USA). All tests were two-sided, and *P* < 0.05 was considered statistically significant.

## Result

### Vision health among male and female students

Table 2 shows the differences in vision health between male and female students. Among all sample students, female students have significantly higher LogMAR scores than male students, indicating worse visual acuity (Table 2, row 1, columns 1 and 2, significant at the 1% level). Differences between males and females were equally significant under different family structures. (Table 2, row 2 and 5, column 1 and 2, significant at the 5% level).

**Table 2:** The differences in vision health between male and female students

Variable	Sample (N)	LogMAR				Visual impairment			
		(1)	(2)		*P*-value	(3)	(4)		*P*-value
		Male	Female	T-test difference		Male	Female	T-test difference	
		mean(SD)	mean(SD)	(2)-(1)		n(%)	n(%)	(4)-(3)	
All sample	19934	0.20(0.27)	0.23(0.28)	0.03	0.000***	2329(23.03)	2547(26.68)	3.65	0.000***
Student is the only child in the family	1939	0.22(0.29)	0.26(0.30)	0.04	0.003**	356(27.70)	214(32.72)	5.02	0.022*
Student is the youngest child in the family	7723	0.19(0.27)	0.23(0.28)	0.04	0.000***	1188(22.75)	655(26.18)	3.43	0.000***
Student is the oldest child in the family	6701	0.20(0.28)	0.24(0.29)	0.04	0.000***	649(22.55)	1113(29.11)	6.56	0.011**
Student is the middle child in the family	3571	0.18(0.25)	0.20(0.28)	0.02	0.012*	199(19.86)	565(21.99)	2.13	0.163

^***^ *p* < 0.001, ** *p* < 0.01, * *p* < 0.05

Of the students, female students have a significantly higher prevalence of visual impairment than male students (Table 2, row 1, columns 3 and 4, significant at the 1% level). The prevalence of visual impairment among male students was 23.03% and 26.68% among female students, which is 3.65% points higher than the male students. The high prevalence of visual impairment in females exists in different family structures (Table 2, row 2 to 4, column 3 and 4, significant at the 5% level). Still, the gender difference in the prevalence of visual impairment is not significant when the student's birth order was the middle child (Table 2, row 5, column 3 and 4).

### Vision exam behavior among male and female students

The results also show that the vision exam rate of female students is lower than that of male students. In the overall sample, the vision exam rate of female students was 16.80% and 18.23% for male students. Female students’ exam rate is about 1.43% points lower, on average, in vision exam rate compared to male students (Table 3, Row 1, significant at the 1% level).

**Table 3:** The differences in vision exam behavior between male and female students

Variable	Sample (N)	Vision exam			
		(1)	(2)	T-test difference	*P* -value
		Male	Female	(2)-(1)	
		n(%)	n(%)		
All Sample	19934	1893(18.23)	1604(16.80)	-1.43	0.001***
Student is the only child in the family	1939	281(21.87)	146(22.32)	0.45	0.819
Student is the youngest child in the family	7723	854(16.36)	409(16.35)	-0.01	0.991
Student is the oldest child in the family	6701	595(20.67)	701(18.34)	-2.33	0.017*
Student is the middle child in the famil	3571	163(16.27)	348(13.55)	-2.72	0.037*

^***^ *p* < 0.001. * *p* < 0.05

We next examine gender differences in vision exam rates among different family structure subgroups. Gender differences in vision exam rate were not significant when the sample student was the only child in the family, or when the sample student was the youngest child in the family (Table 3, Rows 2 and 3). In our sample, when the sample student was the oldest child in the family, the vision exam rate of female students was 18.34% and 20.67% for male students. Female students’ exam rate is about 2.33% points lower, on average, in vision exam rate compared to male students (Table 3, Row 4, significant at the 5% level). When the student’s birth order was the middle child, the vision exam rate of female students was 13.55% and 16.27% for male students. Female students’ exam rate is about 2.72% points lower, on average, in vision exam rate compared to male students (Table 3, Row 5, significant at the 5% level).

We also conduct a multivariate analysis of the gender difference in vision exam rates in different family structures. The results of this analysis are presented in Table 4. Overall, our multivariate analysis confirms the findings of our descriptive analysis. In the whole group, there is a gender difference in vision exam rate, and this difference changes as the family size and birth order. Gender differences in vision exam rate disappeared when the sample students were only children or the youngest children in the family. Gender differences in the frequencies of vision exams exist among children with younger siblings, that is, who were the eldest or the middle children in their families.

**Table 4:** Logistic regression model of gender difference vision exam among 19,934 students in different family structure

Variable	Vision exam				
	All sample	Student is the only child in the family	Student is the youngest child in the family	Student is the oldest child in the family	Student is the middle child in the family
Female	-0.023***	-0.014	-0.008	-0.040***	-0.030*
	(-0.005)	(-0.021)	(-0.009)	(-0.01)	(-0.014)
LogMAR	0.304***	0.327***	0.277***	0.342***	0.261***
	(-0.015)	(-0.035)	(-0.02)	(-0.023)	(-0.031)
Age	0.006	-0.008	0.009*	0.001	0.016*
	(-0.004)	(-0.009)	(-0.005)	(-0.005)	(-0.007)
Math score	0.008	0.018	0.009	0.01	-0.001
	(-0.005)	(-0.01)	(-0.006)	(-0.006)	(-0.008)
Both parents have an education level above high school	0.053***	0.043	0.031*	0.073***	0.032
	(-0.01)	(-0.022)	(-0.015)	(-0.013)	(-0.017)
Left behind children	0.004	0.021	-0.01	0.007	-0.002
	(-0.009)	(-0.026)	(-0.012)	(-0.015)	(-0.019)
Household assets are in the bottom 25%	-0.032***	-0.042	-0.018	-0.041***	-0.038**
	(-0.007)	(-0.022)	(-0.009)	(-0.011)	(-0.012)
Location	-0.016	-0.001	-0.013	-0.014	-0.002
	(-0.019)	(-0.031)	(-0.019)	(-0.022)	(-0.023)
N	19916	1938	7718	6693	3567

Standard errors in parentheses. *** *p* < 0.001, ** *p* < 0.01, * *p* < 0.05

### Eyeglasses own among male and female students

Of the 19,934 students in 252 sample schools who were given vision examinations and surveys, 4,939 (24.3%) students with visual impairment. Only these students are included in the analysis sample in this section. Studies have shown that eyeglasses ownership is highly correlated with students' visual acuity [31, 33, 34]. In this section, we divide students into two sub-samples, those with mild poor vision and those with moderate to severe poor vision, and then explore gender differences in eyeglasses ownership across different family sizes and birth order in these two sample groups.

In the mild poor vision student sample (Table 5, Panel A), female students had lower rates of eyeglasses ownership than male students. In an overall mild poor vision sample, the eyeglasses ownership rate of female students was 6.60% and 9.42% male students. Female students’ eyeglasses ownership rate is about 2.82% points lower, on average, eyeglasses ownership rate compared to male students (Table 5, Row 1, significant at the 1% level).

**Table 5:** The differences in eyeglasses ownership between male and female students

Variable	Sample (N)	Eyeglasses own			
		(1)	(2)	T-test difference	*P*-value
		Male	Female	(2)-(1)	
		n(%)	n(%)		
*Panel A: Mild poor vision*					
All Sample	2965	138(9.42)	99(6.60)	-2.82	0.005**
Student is the only child in the family	332	26(12.32)	5(4.13)	-8.19	0.014*
Student is the youngest child in the family	1135	62(8.30)	21(5.41)	-2.89	0.08
Student is the oldest child in the family	1035	41(10.82)	56(8.54)	-2.28	0.225
Student is the middle child in the family	463	9(7.03)	17(5.07)	-1.96	0.414
*Panel B: Moderate to severe poor vision*					
All Sample	1974	269(29.02)	315(30.09)	1.07	0.604
Student is the only child in the family	238	49(33.79)	23(24.73)	-9.06	0.139
Student is the youngest child in the family	708	115(26.08)	81(30.34)	4.06	0.220
Student is the oldest child in the family	727	89(32.96)	153(33.48)	0.52	0.887
Student is the middle child in the family	301	16(22.54)	58(25.22)	2.68	0.648

** *p* < 0.01, * *p* < 0.05. The analysis sample is visual impairment students

We also examine gender differences in eyeglasses ownership rates among different family structure subgroups. When the student was the only child in the family, the eyeglasses ownership rate of female students was 4.13% and 12.32% of male students. Female students’ eyeglasses ownership rate is about 8.19% points lower, on average, eyeglasses ownership rate compared to male students (Table 5, Row 2, significant at the 5% level). Gender differences in eyeglasses ownership rate were not significant when the sample student had other brothers or sisters (the sample student was the youngest child in the family, or the oldest child in the family or the birth order was the middle child) (Table 5, Row 4 and 5).

In the moderate to severe poor vision student sample (Table 5, Panel B), there is no significant difference between female and male students regarding eyeglasses ownership. There were also no gender differences in student eyeglasses ownership rates under the different family sizes and birth orders.

We also conduct a multivariate analysis of the gender difference in the rate of eyeglasses ownership in different family structures by different visual acuity groups. The results of this analysis are presented in Table 6. Overall, our multivariate analysis corroborates the findings of our descriptive analysis: In the mild poor vision student sample, there was a gender difference in eyeglasses ownership. Gender differences in eyeglasses ownership were observed when the sample students were only children in the home. There was no gender difference in eyeglasses ownership rate whether the sample students were the youngest children, the oldest child in the household, or the middle child in the birth order.

**Table 6:** Logistic regression model of gender difference eyeglasses ownership among 4,939 visual impairment students in different family structures

Variable	Eyeglasses own				
	All Sample	Student is the only child in the family	Student is the youngest child in the family	Student is the oldest child in the family	Student is the middle child in the family
*Panel A: Middle poor vision*					
Female	-0.029***	-0.085**	-0.026	-0.027	-0.029
	-0.01	-0.028	-0.015	-0.019	-0.024
*panel B: Moderate to severe poor vision*					
Female	0.01	-0.115	0.046	0.013	0.003
	-0.02	-0.06	-0.036	-0.037	-0.056
Control variable	YES	YES	YES	YES	YES

^***^ *p* < 0.001, ** *p* < 0.01. Control variables include the student's age, visual acuity, standardized math test score, whether parents have a high school or above education, parental migration status, household asset index and location. The analysis sample is visual impairment students

## Discussion

Nowadays, the visual impairment of rural students in China is still serious and gender inequality still exists; exploring the gender difference in children's visual health in rural areas still has a particular significance. This study examined gender differences in the vision health and behavior of rural Chinese children by family size and birth order, using cross-sectional data from rural China. We first examined gender differences in children's vision health in rural China. We then estimated gender differences in children's vision exam behavior by family size and birth order. Finally, we explored gender differences in children's vision correction behavior (eyeglasses ownership) by family size and birth order.

The results of this study show that female students have worse vision than male students, which is consistent with previous research in rural areas [34]. This study further verifies that gender differences in health outcomes remain robust after controlling for family size and birth order. The study also provides more evidence for gender differences in health outcomes in developing regions [41, 42].

Gender differences in vision health-seeking behaviors (mainly including vision exam behaviors and vision correction behaviors) among rural Chinese children might be one factor for the gender inequalities in vision health outcomes. In terms of vision exam healthcare, overall girls received less than boys. This shows that females in China's rural areas are disadvantaged in terms of healthcare resource allocation [43]. This prejudice, however, varies depending on the girl's birth order and the number of children in the family [44]. On the one hand, whether a child is the family's only child or the youngest child, they are in a relatively advantageous position in the distribution of family resources [45–47], and the gender gap in vision health exam behavior disappears. On the other hand, when children are the eldest or middle child in the birth order (the number of children in the family grows to a minimum of three), they are at a disadvantage in the allocation of family resources, and the gender gap in vision exam behavior widens. This finding implies that when children are disadvantaged in terms of household resource distribution, gender inequality in resource allocation is exacerbated [23, 26].

This study distinguishes between vision examination behavior and vision correction behavior because there is a significant cost difference between the two behaviors. Especially in the sample area of this study. Vision correction behavior requires more family resources and costs [48], and it is impacted by vision severity [37]. Gender differences in vision health and vision-seeking behaviors among children in rural areas are rooted in the allocation of limited family resources; hence, this gap widens when the cost of behavior increases significantly.

Girls in rural China had lower rates of eyeglasses ownership than boys among the mildly vision impaired. At the same time, due to the higher cost of health behaviors, even when children had the advantage of relative family resource allocation (only child), girls still had lower eyeglasses ownership rates than boys, whereas there was no gender difference in vision correction behavior(eyeglasses ownership) for children without the advantage of relative family resource allocation, this result was due to a decrease in eyeglasses ownership for boys due to limited family resources.

There was no difference in the rate of vision correction between boys and girls in the group of children with moderate to severe visual impairment, except for one-child families. The severity of vision impairment might explain this finding. The fact that girls had poorer vision than boys indicates that girls experienced more severe physical, mental, academic, and life difficulties due to vision problems than boys [31], and only under these conditions can their health problems be solved relatively equally, which also confirms the disadvantageous position of girls in the utilization of family resources.

Furthermore, the findings of this study suggest that overall vision health-seeking behaviors among boys and girls in rural China are extremely low, with less than 30% of children getting vision exams and only around 17% of children with vision difficulties having eyeglasses. These findings imply that, while focusing on gender differences in children's vision health behaviors in low-income regions is essential, more policies and measures are needed in rural China to improve children's overall vision health behaviors.

This study has several strengths. First, in particular, most studies on health service uptake rely on self-reported data. Instead, this study examined the actual intake of vision health services (eyeglasses ownership). Second, this study examines changes in gender differences in children's vision health and behavior when changes in children's birth order, and family size give children a different advantage in the distribution of family resources.

Several limitations should be acknowledged when considering our results. First, the study participants were all from western China, thus generalizing the findings should be done with caution. Secondly, this study data is collected at 2012. Although these data are relatively old, recent studies in rural China still found high rates of visual impairment, low correction rates [49, 50], and gender gaps in health and health behaviors [10, 11], suggesting that the situation in rural China is much the same as in 2012. Therefore, we have reason to believe that our findings and conclusions are still applicable to rural China today. Third, self-reported recall data about the other variables, as adopted by most studies of visual acuity [32, 51, 52], depend on the reliability of informants; this issue may be greater when younger children are involved, as in the present case. Given the resource limitations to researchers following a large cohort of young children, the self-reported recall was determined to be the best method for our study’s visual acuity data collection.

Despite these limitations, this study contributes to a growing body of literature suggesting that discrimination against girls exists in the investment of resources in vision health in rural China and the change of family size and birth order does not eliminate this gender discrimination when the cost of health behaviors rises, resulting in gender differences in children's vision health in rural China.

Poor visual health and uncorrected vision problems have substantial financial implications for affected individuals, families, and communities. Conservative assessments based on the latest prevalence figures for 2020 suggest that annual global productivity loss from vision impairment is approximately US$410.7 billion purchasing power parity [53]. Studies have shown that visual impairment has negative effects on school-aged children’s academic performance [31], physical and mental health and quality of life [54]. Gender differences in vision health behaviors among rural children can further negatively impact girls' future vision health. Therefore, if future policies are not adopted to improve gender differences in children’s vision health-seeking behaviors in rural areas, the future gap in human capital accumulation between men and women is likely to worsen.

## Conclusion

Our analysis of children's vision health and behavior in rural areas contributes another layer of understanding to the gender gap in healthcare in rural China. Overall, there is a gender difference in vision health outcomes among rural Chinese children, and this imbalance is related to the gender difference in rural children's vision health-seeking habits. Gender differences in vision health behaviors change depending on the child's birth order and the number of children in the family. Gender differences in vision health behaviors are reduced when the child is the only child in the family or the youngest child. Gender discrimination in resource allocation increases further when the child is the oldest in the family or the middle child in the birth order (the number of children in the family increases). When the cost of health behaviors rises, however, the changes in family size and birth order will not be enough to compensate for gender disparities in health behaviors.

Gender differences in children’s vision health-seeking behaviors in rural China reflect inequities in family health investments, and this inequity can lead to a long-term negative impact on girls’ vision health behaviors, resulting in gender differences in vision health outcomes between boys and girls, and ultimately gender disparities in human capital accumulation. This discrepancy is unfair and should be reversed in time to ensure that children receive equitable investment in vision health resources.

The reason for the gender gap in visual health behavior is the long-standing preference for boys in China, which underestimates the benefits of investing in girls and therefore prioritizes investing in boys when household resources are limited [16–22]. Future policies could consider providing medical subsidies to reduce the financial cost of vision health behaviors and alleviate the gender gap in vision health behaviors. On the other hand, carry out information interventions to promote gender equality by preaching the importance of girls’ acceptance of vision health behaviors, that girls are not less valuable than boys in the labor market, etc., to correct families’ misconceptions about the value of girls and to fundamentally gender bias in families.

## Data Availability

The dataset used and/or analyzed during the current study are available from the corresponding author on reasonable request.

## Abbreviations

ETDRS: Early Treatment Diabetic Retinopathy Study
MAR: Minimum Angle of Resolution
